# Trade‐off drives Pareto optimality of within‐ and among‐year emergence timing in response to increasing aridity

**DOI:** 10.1111/eva.13145

**Published:** 2020-11-06

**Authors:** Joseph Waterton, Susan J. Mazer, Justin R. Meyer, Elsa E. Cleland

**Affiliations:** ^1^ Ecology, Behavior & Evolution Section University of California San Diego La Jolla CA USA; ^2^ Department of Ecology, Evolution and Marine Biology University of California Santa Barbara Santa Barbara CA USA

**Keywords:** adaptation, aridity, bet‐hedging, climate change, emergence timing, pareto optimality, phenology, trade‐off

## Abstract

Adaptation to current and future climates can be constrained by trade‐offs between fitness‐related traits. Early seedling emergence often enhances plant fitness in seasonal environments, but if earlier emergence in response to seasonal cues is genetically correlated with lower potential to spread emergence among years (i.e., bet‐hedging), then this functional trade‐off could constrain adaptive evolution. Consequently, selection favoring both earlier within‐year emergence and greater spread of emergence among years—as is expected in more arid environments—may constrain adaptive responses to trait value combinations at which a performance gain in either function (i.e., evolving earlier within‐ or greater among‐year emergence) generates a performance loss in the other. All such trait value combinations that cannot be improved for both functions simultaneously are described as Pareto optimal and together constitute the Pareto front. To investigate how this potential emergence timing trade‐off might constrain adaptation to increasing aridity, we sourced seeds of two grasses, *Stipa pulchra* and *Bromus diandrus*, from multiple maternal lines within populations across an aridity gradient in California and examined their performance in a greenhouse experiment. We monitored emergence and assayed ungerminated seeds for viability to determine seed persistence, a metric of potential among‐year emergence spread. In both species, maternal lines with larger fractions of persistent seeds emerged later, indicating a trade‐off between within‐year emergence speed and potential among‐year emergence spread. In both species, populations on the Pareto front for both earlier emergence and larger seed persistence fraction occupied significantly more arid sites than populations off the Pareto front, consistent with the hypothesis that more arid sites impose the strongest selection for earlier within‐year emergence and greater among‐year emergence spread. Our results provide an example of how evaluating genetically based correlations within populations and applying Pareto optimality among populations can be used to detect evolutionary constraints and adaptation across environmental gradients.

## 1 INTRODUCTION

Plant populations provide some of the best examples of local adaptation to climatic conditions (e.g., Colautti & Barrett, 2013; Exposito‐Alonso et al., 2018; Fournier‐Level et al., 2011; Wadgymar et al., 2017), and anthropogenic climate change is expected to further require species to evolve in order to persist in novel conditions (Hoffmann & Sgro, 2011; Jump & Peñuelas, 2005). However, adaptive evolution can be constrained by many factors, including trade‐offs between fitness‐related traits (Etterson & Shaw, 2001). Characterizing trade‐offs that constrain potential adaptive responses is therefore important for understanding how plant populations adapt to current and future climate. Previous studies have revealed such trade‐offs by directly measuring selection on correlated traits and showing that the direction of selection is antagonistic to the direction of the correlation between traits (e.g., Caruso, 2004; Etterson & Shaw, 2001). However, such an approach is particularly challenging when the adaptive value of a trait manifests over many years, or for long‐lived species in which estimates of lifetime fitness are difficult to obtain.

Following dispersal, the timing of seedling emergence determines the environmental conditions experienced by plants, strongly influencing fitness as well as patterns of selection on traits expressed later in development (reviewed in Donohue et al., 2010). As a result, emergence timing is a key trait influencing adaptation to local conditions as well as potential adaptation in response to anthropogenic climate change (Cochrane et al., 2015; Donohue et al., 2010; Walck et al., 2011). In seasonal ecosystems, the timing of emergence can be viewed as a complex trait composed of two potentially independent traits affecting fitness through distinct life history functions: (a) within‐year emergence time in response to seasonal cues (i.e., emergence speed); (b) among‐year emergence spread (defined here as the fraction of seeds persisting in the seed bank among years). Considering emergence timing within a given year, emerging earlier in response to seasonal germination cues is often associated with increased fitness, resulting from longer windows for growth and reproduction as well as the potential to preempt resources and suppress the growth of late arrivers (Verdú & Traveset, 2005). However, a number of factors could selectively favor later emergence in response to the onset of seasonal germination cues. For example, earlier emergence may increase the risk of growing before the onset of reliably tolerable conditions, for example by exposing individuals to a prolonged dry period (Wainwright et al., 2012) or a late frost (Skálová et al., 2011). Additionally, earlier emergence can increase susceptibility to mammalian herbivores, potentially through increased apparency (Waterton & Cleland, 2016). Considering emergence timing over multiple years, more variable environments that result in relatively high variance in fitness among years favor greater spreading of emergence (maximizing geometric mean fitness, a form of bet‐hedging) (Gremer et al., 2016; Tielbörger et al., 2012). Spreading emergence among years requires that: (a) not all seeds produced each year germinate, and (b) some ungerminated seeds survive. These together determine the fraction of seeds that persist between years, a measure of potential among‐year emergence spread.

A functional trade‐off between the speed of emergence within years and the potential to spread emergence among years may constrain the range of possible trait combinations that can evolve in plant populations. Dormancy prevents seeds from germinating in conditions that would otherwise be sufficient; genetically based dormancy may therefore have pleiotropic effects on both delaying emergence and increasing the number of ungerminated seeds (Bewley et al., 2013; Long et al., 2015). Supporting this inference, quantitative trait loci (QTLs) that influence primary dormancy have been shown to colocate with QTLs affecting both germination fraction and within‐year emergence time under field conditions, with increased dormancy associated with lower germination fractions and later emergence (Huang et al., 2010). Additionally, dormancy may further promote among‐year emergence spread by increasing the resistance of seeds to aging in soils compared to non‐dormant seeds (reviewed in Long et al., 2015). Non‐dormant seeds can also persist across years if germination cues (e.g., water, light, and temperature) are not met (Long et al., 2015), and more stringent (genetically based) cue requirements in non‐dormant seeds may result in the pleiotropic effects of lowering overall germination fractions and delaying germination among seeds that do germinate (Bewley et al., 2013). Thus, due to pleiotropy or genetic linkage, we expect the fraction of persistent seeds (i.e., potential spread of emergence across years) to be positively associated with average days to emergence in response to seasonal cues in any given year (i.e., within‐year emergence speed), impeding the independent evolution of these traits. Consistent with this potential constraint on adaptive evolution within species, it has been shown that, across different species occupying similar habitats, earlier emergence is associated with lower soil seed persistence (Saatkamp et al., 2011). While genetic linkage that leads to associations between emergence time and seed persistence can be broken over time through recombination, the patterns cited above suggest that the trade‐off between within‐year emergence speed and among‐year emergence spread may be commonly expressed.

**Figure 1:**
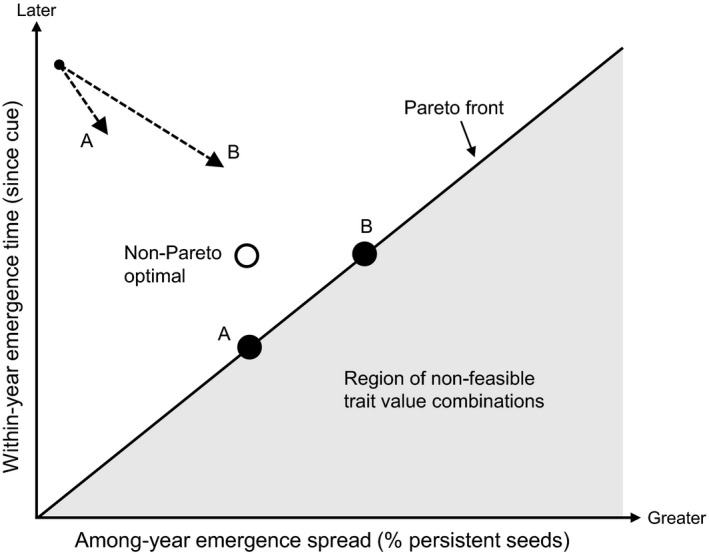
Hypothesized constraint to the evolution of both earlier within‐year emergence and greater among‐year emergence spread resulting from a trade‐off between the two traits. Dashed arrows are vectors representing the relative strength of selection for earlier within‐year emergence and greater among‐year emergence spread in environments *A* and *B*, and black circles represent the corresponding trait values that evolve. Non‐feasible trait combinations resulting from a trade‐off between the two traits are represented by the gray shaded area. Adaptive responses are constrained to Pareto optimal trait combinations at which both functions (earlier within‐year emergence and greater among‐year emergence spread) cannot be simultaneously improved. The set of Pareto optimal trait value combinations, or Pareto front, is not limited to forming a straight line as depicted here. In this example, each environment results in the evolution of trait value combinations on the Pareto front, but environment *B*, in which there is stronger selection for earlier within‐year emergence, results in the evolution of later emergence than environment *A*, which exerts weaker selection for earlier emergence

In scenarios where selection favors both earlier within‐year emergence (for earlier growth) and greater among‐year emergence spread (for greater bet‐hedging), a trade‐off between these traits will prevent plant populations from optimizing both functions simultaneously. Adaptive responses will instead be bounded by combinations of trait values for which a performance gain in one function (i.e., the evolution of either earlier within‐year emergence or greater among‐year emergence spread) can only be achieved with a performance loss in the other (Figure 1). All such trait value combinations that cannot be improved for all functions simultaneously are described as Pareto optimal and together constitute the Pareto front. The Pareto front concept originates from the fields of economics and engineering but has more recently been applied to biological phenotypes (e.g., Sheftel et al., 2013; Shoval et al., 2012). For the two emergence timing functions that we describe, this is the set of phenotypes for which no others have both earlier within‐year emergence and greater among‐year emergence spread. Note that the Pareto front is in reference to performance in a set of functions and thus does not reflect overall fitness. For two traits, such as within‐ and among‐year emergence timing, that each determines performance in separate fitness‐related functions (i.e., early growth and bet‐hedging), the Pareto front is analogous to a two‐trait trade‐off curve. However, Pareto optimality can also be evaluated for more than two fitness‐related functions, each of which can be influenced by multiple traits (see Sheftel et al., 2013; Shoval et al., 2012). Also note that while the hypothetical Pareto front in Figure 1 is depicted as a straight line, Pareto fronts are not limited to this shape. The specific combinations of trait values that evolve along a Pareto front will largely depend on the relative fitness contributions of each function (i.e., the relative strength of selection, Figure 1), but can also be influenced by the underlying genetics of traits that constrain the shape of the Pareto front itself (Maharjan et al., 2013). Furthermore, phenotypic plasticity can alter the expression genetically based correlations between traits (Stearns et al., 1991) and could there thus influence emergence timing trait values that occupy the Pareto front.

An important potential consequence of the hypothesized trade‐off is that environments that select more strongly for either earlier within‐year emergence or greater among‐year emergence spread could result in trait values that are further from their optimum when considering individual traits, but are in fact on the Pareto front when considering trait combinations (Figure 1). Directly measuring selection to demonstrate the constrained evolution of within‐ and among‐year emergence timing is challenging because the adaptive value of among‐year emergence spread is determined over many years or decades, and selection on within‐year emergence can fluctuate between years (Kalisz, 1986). Indirect evidence of historical adaptation can instead be obtained by studying traits along environmental gradients (Pratt & Mooney, 2013). However, as shown by the example in Figure 1, for correlated traits that affect fitness through separate life history functions, measuring only a single trait—such as within‐year emergence time—across an environmental gradient could result in erroneous inferences about patterns of historical selection. Instead, significant associations between environmental variables and the Pareto optimality of trait combinations (within a given sample of populations) could provide indirect evidence of constrained evolutionary responses.

Aridity gradients in Mediterranean climate regions are ideal for investigating a potential trade‐off between within‐year emergence speed and among‐year emergence spread. In such regions, water availability is the major control over seasonal plant growth and is a key factor shaping the evolution of emergence timing within and among years (Arroyo et al., 2006; Petrů & Tielbörger, 2008; Torres‐Martínez et al., 2017). Plant populations toward the drier ends of aridity gradients tend to experience shorter windows of favorable environmental conditions (Aviad et al., 2004; Metz et al., 2020) as well as greater interannual variability in conditions than populations occupying more mesic sites (Davidowitz, 2002; Metz et al., 2020). As a result, more arid sites might select for earlier emergence within years to facilitate rapid growth (Dickman et al., 2019; Sexton et al., 2011), greater spread of emergence among years as a way of bet‐hedging (Arroyo et al., 2006; Petrů & Tielbörger, 2008; Venable & Brown, 1988), or both, which could lead to constrained adaptive evolution. Examining how traits vary along aridity gradients is particularly important because it provides insights into adaptive responses to climatic conditions which are consistent with the direction of climate change (Pratt & Mooney, 2013). That is, adaptive responses to spatial variation in aridity may serve as a proxy for—and facilitate predictions regarding—adaptive responses to upcoming temporal variation in aridity predicted by climate models. Globally, many Mediterranean ecosystems are projected to become increasingly arid, with warmer and drier average conditions as well as increased interannual variability in precipitation (Alpert et al., 2008; Berg & Hall, 2015; IPCC, 2013; Seager et al., 2007; Yoon et al., 2015).

Emergence timing is highly dependent on environmental cues experienced by seeds in the soil (Bewley et al., 2013), and this phenotypic plasticity is expected to play a key role in determining population persistence under climate change (Walck et al., 2011). Variation in environmental conditions can shift both trait values and the trait values favored by selection (i.e., phenotypic optima), and plasticity that shifts emergence timing trait values toward the phenotypic optima that can be predicted by cues in a given year represents a form of predictive plasticity (Gremer et al., 2016). Such predictive plasticity could therefore reduce fitness costs associated with the proposed evolutionary constraint imposed by a trade‐off between within‐year emergence speed and among‐year emergence spread. For example, if lower soil moisture predicts less favorable growing conditions, thus shifting the pengiredicted phenotypic optimum toward higher seed persistence, this could promote seed persistence by decreasing the proportion of seeds that germinate (Bewley et al., 2013) or increasing the survival of non‐germinating seeds (Long et al., 2015; Mordecai, 2012). Such plastic responses of emergence timing traits to water availability are consistent with predictive plasticity if they match clinal patterns of trait variation across an aridity gradient.

We carried out a greenhouse experiment to investigate the potential for a trade‐off between within‐year emergence speed and among‐year emergence spread to constrain adaptive responses to aridity in two widespread California grasses, the native perennial *Stipa pulchra* (Hitchc.) Barkworth and the exotic annual *Bromus diandrus* (Roth). We also imposed two watering treatments to investigate how plasticity in response to drier conditions might alter the fitness costs associated with such an evolutionary constraint. We hypothesized that: (a) among genotypes, earlier emergence within years is associated with lower potential to spread emergence among years; (b) based on geographic patterns of trait variation among populations, selection for earlier within‐year emergence and greater among‐year emergence spread is stronger in more arid environments, but the evolution of both early within‐year emergence and greater among‐year emergence spread is constrained; (c) plasticity in emergence timing traits in response to water availability can alter the fitness costs associated with the evolutionary constraint generated by the trade‐off between within‐year emergence speed and among‐year emergence spread.

## 2 MATERIALS AND METHODS

### 2.1 Study system

Coastal California is characterized by a steep gradient in aridity that is consistent with projections of future climate change in the region, with southern regions tending to be warmer and drier, but with greater interannual variability in precipitation, than northern regions (Pratt & Mooney, 2013). Since European settlement in the 18th century, exotic annual grasses have become dominant in California, displacing much of the native flora (Heady, 1977). The two widespread grasses used in this study, the native perennial *Stipa pulchra* and the exotic annual *Bromus diandrus*, are therefore representative of two key functional groups in California grasslands that differ with respect to origin and life history strategy. Both focal species emerge predominantly in response to the onset of winter rains (Bartolome & Gemmill, 1981; Marañón & Bartolome, 1989).

*Stipa pulchra* (purple needlegrass) is a native perennial bunchgrass found in woodland, chaparral, and grassland from Baja California to northern California (Baldwin et al., 2012). *S. pulchra* can be long‐lived, with some individuals able to survive for over 100 years (Hamilton et al., 2002). A study of neutral genetic markers shows that *S. pulchra* harbors relatively low genetic variation within populations but high genetic differentiation among populations, likely due to high rates of self‐fertilization (reported selfing rates ≈ 1) (Larson et al., 2001). Consistent with this, quantitative traits in *S. pulchra* show evidence of ecotypic differentiation among populations (Knapp & Rice, 1998), although no studies have assessed both within‐year and among‐year emergence timing. *S. pulchra* is characterized by high seed viability, with studies recording percentages of 90% or greater (Deering & Young, 2006; Dyer et al., 2000). However, a previous study in northern California found low persistence of *S. pulchra* seeds in the soil among years (Bartolome & Gemmill, 1981).

*Bromus diandrus* (great brome or ripgut brome) is an exotic annual grass species native to Eurasia. This species is found in many habitats across California and is particularly dominant in disturbed areas, such as abandoned agricultural fields (Stromberg & Griffin, 1996). *B. diandrus* is also largely self‐fertilizing (reported selfing rates > 0.99) (Kon & Blacklow, 1990). Similar to *S. pulchra*, *B. diandrus* is characterized by high seed viability, with studies recording percentages over 90% (Deering & Young, 2006; Harradine, 1986; Kleemann & Gill, 2013). Studies of *B. diandrus* in southern Australia have found that dormancy levels can vary greatly among populations, with dormancy being lost over time through after‐ripening and also by exposure to cold temperatures (Kleemann & Gill, 2013). *B. diandrus* germination is strongly inhibited by light (Kleemann & Gill, 2013).

### 2.2 Source populations and field sampling

**Figure 2:**
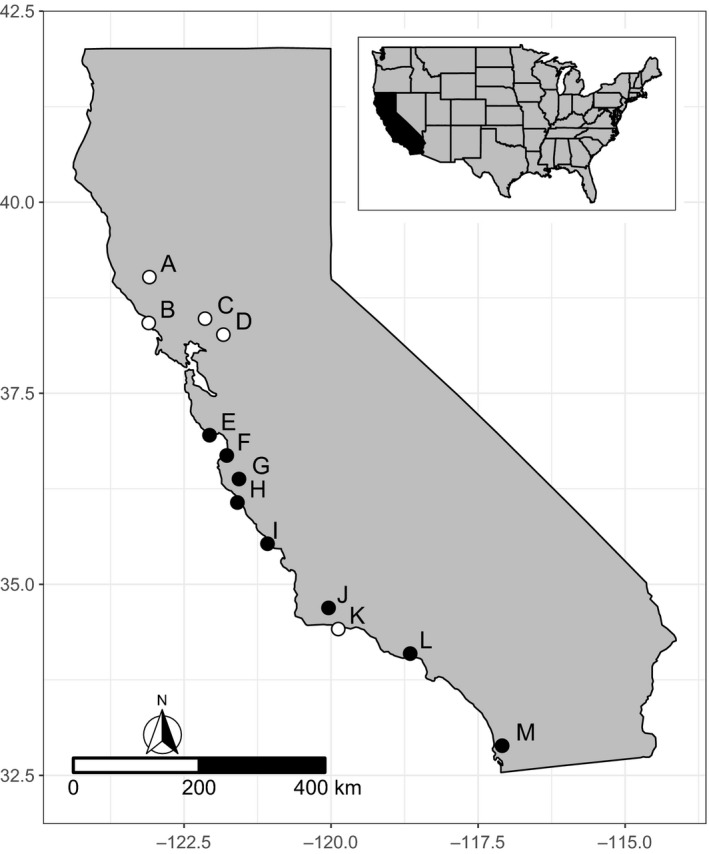
*Stipa pulchra* and *Bromus diandrus* source populations. *S. pulchra* was collected from all thirteen sites. Filled circles represent sites where *S. pulchra* and *B. diandrus* were collected. (A) Hopland Research and Extension Center; (B) Bodega Marine Reserve; (C) Quail Ridge Reserve; (D) Jepson Prairie Reserve; (E) Younger Lagoon Reserve; (F) Fort Ord Natural Reserve; (G) Hastings Natural History Reservation; (H) Landels‐Hill Big Creek Reserve; (I) Kenneth S. Norris Rancho Marino Reserve; (J) Sedgwick Reserve; (K) Coal Oil Point Natural Reserve; (L) Stunt Ranch Santa Monica Mountains Reserve; and (M) Elliott Chaparral Reserve

In April 2015, we collected seeds of *S. pulchra* from 13 populations and *B. diandrus* from 8 populations (Figure 2). At each site, we collected seeds from 20 plants (hereafter referred to as maternal lines) situated in open flat areas and spaced at least 5 m apart. We stored seeds at ambient temperatures for 2 weeks and then at 4 °C until planting.

Historical climatic conditions shape plant adaptation across environmental gradients (Colautti & Barrett, 2013; Exposito‐Alonso et al., 2018; Fournier‐Level et al., 2011; Wadgymar et al., 2017). Meanwhile, deviations of climate variables from long‐term means, or “anomalies,” can cause plastic shifts in plant traits including phenology, reproductive output, and seed mass (Bontrager & Angert, 2016; Mazer et al., 2020; Munson & Sher, 2015). Therefore, for each site we quantified: (a) historical mean aridity; (b) the deviation of aridity in the year of seed collection from the historical mean. To quantify historical mean aridity, we calculated the unitless aridity index (AI), the ratio of mean annual precipitation to mean annual potential evapotranspiration (P/PET) (Malmström, 1969), for the years 1985 – 2014 (hereafter “historical AI”). Historical AI values that are closer to zero indicate greater average aridity than more positive values. As expected, historical AI was positively correlated with latitude (*r* = 0.69). We quantified the deviation of aridity in the year of seed collection from the historical mean as the difference between the year of seed collection AI (annual P/PET for May 2014–April 2015) and the historical AI (hereafter “deviation AI”). Negative deviation AI values indicate that the year of collection was drier than the historical average, while positive values indicate a wetter than average collection year. We retrieved temperature and precipitation data for calculating AIs from the PRISM Climate Group database (prism.oregonstate.edu/). We estimated potential evapotranspiration using temperature and latitude data with the Thornthwaite equation (Thornthwaite, 1948), in the R package *SPEI* (Beguería & Vicente‐Serrano, 2017). Climate summaries for source populations are provided in Table S1.

### 2.3 Greenhouse experiment

The experiment was conducted at the University of California San Diego Biology Field Station greenhouses (32.885°N, 117.230°W) between March and August 2016. We note that this represents a later seasonal start of emergence and growth of the two focal species, and this was due to the timing of greenhouse availability. While dormancy cycling can be important for controlling germination across seasons (Edwards et al., 2017), a previous growth chamber experiment manipulating day length, soil moisture, and temperature showed favorable germination of *S. pulchra* as well as exotic annuals (not *B. diandrus*, but the congener *B. hordeaceus* and others) outside of the growing season (Wainwright & Cleland, 2013). For every maternal line in each source population, we randomly chose six *S. pulchra* seeds and five *B. diandrus* seeds to plant in each of two watering treatments, “high” and “low,” for a total of 1,600 *B. diandrus* seeds (8 populations × 20 maternal lines × 2 watering treatments × 5 seeds per treatment) and 3,120 *S. pulchra* seeds (13 populations × 20 maternal lines × 2 watering treatments × 6 seeds per treatment). We weighed seeds individually with awns attached, avoiding any that appeared empty or non‐viable.

We planted seeds individually to a depth of 1 cm, with radicles oriented downwards, into RLC4 “cone‐tainers” (Stuewe & Sons, Inc., Tangent OR) filled with dry 70/30 topsoil (Agriservice, Inc., Oceanside, CA), a mix of 70% sandy loam soil with 30% humic compost (pH ≈ 7.5). We chose this depth because it is favorable to *S. pulchra* germination (Tilley et al., 2009) and because *B. diandrus* germination is inhibited by light (Kleemann & Gill, 2013). For each species, we arranged cone‐tainers so that each rack contained one seed from every maternal line, with 6 racks per watering treatment for *S. pulchra* and 5 racks per watering treatment for *B. diandrus*. All water was delivered by overhead irrigation. We planted seeds into dry soil to allow all seeds the opportunity to initiate germination simultaneously when water was eventually applied. We first planted *S. pulchra* seeds over several days until 1 March when watering began (Day 0 for *S. pulchra*). We later planted *B. diandrus* seeds over several days until 17 March when watering began (Day 0 for *B. diandrus*).

We watered seeds of both species until soil saturation on their respective Days 0 and 2 to simulate large early season rain events and subsequently imposed the separate watering treatments on Day 4. The high watering treatment received 3 times as much water as the low treatment, which approximately represents the difference in mean annual precipitation between the wettest and driest source populations (Table S1). Seeds in the low watering treatment initially received 10mm of water every four days. Seeds in the high watering treatment received the same 10mm pulse every four days plus an additional 20mm delivered two days after each 10mm pulse. For both species, we doubled the amount of water supplied in each pulse for both treatments beginning 22 April to compensate for warming greenhouse conditions. We rotated cone‐tainer racks every 4 days to account for potential spatial variation in greenhouse conditions. All cone‐tainers received ambient light throughout the experiment. Temperature data inside the greenhouse were unavailable during the experiment; however, the mean temperature at the study site for the duration of the experiment was 17.7 °C (PRISM). Subsequent measurements for a comparable period in 2019 showed that temperatures inside the greenhouse are on average 1 °C warmer than outside (personal observation), and thus, plants experienced temperatures closer to the warmest source populations (Table S1). Mean temperatures for October, during which widespread emergence often occurs in California grasslands (Bartolome, 1979; Young et al., 1981), ranged between 14.4 °C and 19.9 °C in the source populations (PRISM).

We monitored cone‐tainers daily and recorded the date of emergence for each individual. Total emergence of *B. diandrus* was low until Day 10 (< 4% of seeds planted), likely due to drying soils. Therefore, beginning on Day 10, we watered *B. diandrus* cone‐tainers until soil saturation for four consecutive days before restarting the separate watering treatments. We retrieved non‐emerged seeds from the soil over several consecutive days beginning on Days 139 and 141 for *S. pulchra* and *B. diandrus*, respectively. To facilitate the retrieval of seeds, we watered daily during this collection period to soften soils. We rinsed intact seeds with ethanol to surface sterilize them, allowed them to air‐dry, and stored them in coin envelopes at 4 °C until they were scored for viability in July 2017 using a tetrazolium assay (AOSA/SCST, 2010). Viable seeds were scored as persistent, and all non‐viable seeds were scored as having suffered mortality (we acknowledge that some seeds may have been non‐viable at the time of planting). Additionally, because of the increased frequency of watering during seed collection, seedlings that emerged at this time were also scored as persistent.

### 2.4 Statistical analyses

We conducted all statistical analyses separately for each focal species, using R version 3.6.1 (R Core Team, 2019). For *S. pulchra*, we calculated days to emergence from the first watering pulse on Day 0. Due to the low total emergence of *B. diandrus* in response to the initial watering pulses (< 4% of seeds planted), for this species we calculated emergence time from the start of the consecutive‐day watering pulses that began on Day 10. We assigned the earliest emergence time that we observed in the initial low emergence cohort, 4 days, to all individuals that emerged before Day 14 as we assumed that these had initiated germination prior to the start of the consecutive‐day watering pulses on Day 10. We note that this adjustment for *B. diandrus* emergence time did not qualitatively change our results. In both species, emergence time was right‐skewed and therefore square‐root‐transformed to improve normality of residuals (Simons & Johnston, 2006).

Because *S. pulchra* and *B. diandrus* are characterized by high seed viability (≥ 90%) (Deering & Young, 2006; Dyer et al., 2000; Harradine, 1986; Kleemann & Gill, 2013), we inferred that maternal lines with low seed viability were collected prior to the date required for seed maturation. Therefore, to minimize the influence of such maternal lines with low initial seed viability, we excluded from analyses those maternal lines in which, across both watering treatments, fewer than 50% of planted seeds either emerged or persisted (i.e., were “viable”). We also excluded source populations with fewer than 10 maternal lines meeting this viability threshold to exclude those likely collected before their seeds were mature and to ensure reasonable within‐population sample sizes. No *B. diandrus* source populations were excluded, but 10 maternal lines were excluded in total, leaving 150 maternal lines in the analyses reported here (see Table S2 for the numbers of maternal lines meeting the viability threshold in each source population). For *S. pulchra*, the following 5 source populations were excluded entirely: Fort Ord, Hastings, Hopland, Jepson, and Younger Lagoon (Table S2). The mean historical AIs for the *S. pulchra* source populations that were retained (*n* = 8) and excluded (*n* = 5) for analyses were 0.87 and 0.89, respectively (site values ranged between 0.38 and 1.59, Table S1). A total of 17 maternal lines were excluded from the remaining 8 *S. pulchra* populations, leaving 143 in the analyses reported here (Table S2). We note that including *S. pulchra* maternal lines with ≥ 50% viability from the five excluded populations did not qualitatively change our results.

We tested for the influence of population, watering treatment, population × watering treatment interaction, and seed mass on each possible outcome for individual seeds (e.g., emergence time and persistence), using linear mixed models (LMMs) and generalized linear mixed models (GLMMs) with maternal line included as a random effect in all models. Significant watering effect terms indicate plasticity in emergence timing traits, and a significant population × watering treatment interaction indicates that plastic responses differ among source populations. We determined whether plastic responses to watering were consistent with predictive plasticity by comparing their direction to patterns of clinal trait variation (see below). We included seed mass as a covariate to account for potential effects of maternal provisioning. We fit LMMs to test the effect of each factor on emergence time for seeds that emerged (“emergence time”). We fit GLMMs with binomial error distributions and logit link functions to test the effect of each factor on the probability of seed persistence (“persistence”). Because persistence is dependent on both germination and mortality in non‐emerging seeds, we fit separate GLMMs to test the effect of each factor on the probability of emerging (“emergence”) and the probability of seed mortality (“mortality”).

To test for a trade‐off between emergence time and seed persistence fraction in each species, we fit LMMs in which, across both watering treatments, mean emergence time in maternal lines was predicted by the fraction of viable persistent seeds in maternal lines, with source population treated as a random effect. We calculated mean emergence time and seed persistence fraction across watering treatments to maximize sample sizes within maternal lines and to maintain independent observations. Therefore, we did not evaluate how plasticity in response to watering treatment influenced the expression of the trade‐off. Where the fraction of persistent seeds in maternal lines was a significant predictor of mean emergence time, we tested whether this relationship occurred within populations or whether it was driven by covariation of population means. For this, we calculated for each maternal line the deviations in mean emergence time and seed persistence fraction from their respective source population means by subtracting mean source population values from maternal line values. We then fit the same LMM as before, replacing the raw maternal line values with the deviations from their source population means. A significant positive relationship between deviation values indicates that larger fractions of persistent seeds predict longer mean time to emergence independently of differences in mean values among source populations. Additionally, where the fraction of persistent seeds in maternal lines was a significant predictor of mean emergence time, we tested whether seed mass was mediating the relationship by replacing raw emergence time values in the original LMM with the residuals from a regression of mean emergence time against mean seed mass in maternal lines. One limitation of these analyses is that maternal lines in which 100% of living seeds persisted in the soil cannot be included as they have no associated emergence time; however, this occurred only in two maternal lines of *B. diandrus*. We specified LMMs using the R package *nlme* (Pinheiro, Bates, DebRoy, Sarkar, & R Core Team, 2019) and evaluated the significance of fixed effects with Type II tests using the *car* package (Fox & Weisberg, 2011). We specified GLMMs using the R package *GLMMadaptive* (Rizopoulos, 2019) and evaluated the significance of fixed effects with likelihood ratio tests.

Where source population was a significant source of variation in emergence time and the probability of seed persistence, we tested for associations between mean population trait values (individually and in combination) and each AI (historical and deviation). Significant associations with historical AI are consistent with adaptive evolution to aridity. Significant associations with deviation AI indicate that aridity anomalies (higher or lower than historical means) experienced by maternal plants in the year of collection influence seed behavior. We used linear regressions to test the relationships between AIs and source population means for each trait individually, averaged across both watering treatments. We determined the populations on the Pareto front for combinations of earlier emergence and larger seed persistence fraction (i.e., the set of populations for which no other single population possesses both earlier emergence and larger seed persistence fraction) and tested whether aridity was significantly different in populations on versus off the Pareto front. We emphasize that populations are described as being on the Pareto front in the context of our sampled source populations and may not represent populations on the global Pareto front. Furthermore, we emphasize that we consider such populations to be on the Pareto front only with respect to earlier emergence and larger seed persistence fraction. We determined the populations on the Pareto front algorithmically using the *psel* function in the R package *rPref* (Roocks, 2016), with the preference object (i.e., the predetermined direction of optimality) set to simultaneously optimize for earlier emergence and larger seed persistence fraction. We weighted both traits equally in the algorithm because we had no *a priori* hypothesis concerning their relative contributions to fitness. We note that in this case, the Pareto front can also be visually determined (e.g., by inspecting a scatter plot). We tested whether populations on the Pareto front were more historically arid than populations off the Pareto front using one‐tailed two‐sample permutation tests with 10,000 repeats. We had no *a priori* hypothesis of how deviation AI would influence the Pareto optimality of emergence timing traits, so we tested whether this was significantly different in populations on versus off the Pareto front using two‐tailed two‐sample permutation tests with 10,000 repeats.

## 3 RESULTS

### 3.1 Influence of seed characteristics and watering on emergence timing traits

Source population and seed mass were significant predictors of emergence time and seed persistence in both focal species. On average across all source populations, *S. pulchra* and *B. diandrus* emerged 10.2 days and 21.7 days after initial watering pulses, respectively. In both species, emergence time differed significantly among populations (*S. pulchra*: *p* < 0.001; *B. diandrus*: *p* < 0.001; Table 1). Emergence time also decreased with seed mass in both species (*S. pulchra*: *p* < 0.001; *B. diandrus*: *p* < 0.001; Table 1). Across all source populations, 2.6% of *S. pulchra* seeds persisted in total and 85% of maternal lines had 0 persistent seeds. In *B. diandrus*, 19% of seeds persisted and 41% of maternal lines had 0 persistent seeds. In both species, the probability of seed persistence differed significantly among populations (*S. pulchra*: *p* < 0.001; *B. diandrus*: *p* < 0.001; Table 2). Larger seeds were less likely to persist in both species (*S. pulchra*: *p* = 0.012; *B. diandrus*: *p* = 0.044; Table 2), resulting from a higher probability of emergence (*S. pulchra*: *p* < 0.001; *B. diandrus*: *p* < 0.001; Table 2) and despite a lower probability of mortality (*S. pulchra*: *p* < 0.001; *B. diandrus*: *p* < 0.001; Table 2).

**Table 1:** Results of LMMs evaluating the effects of population, watering treatment, population × watering treatment interaction, and seed mass on individual seed emergence time in *Stipa pulchra* and *Bromus diandrus*

Emergence time	Population			Watering treatment				Population × watering treatment			Seed mass			
	*df*	χ^2^	*p*	*df*	χ^2^	*p*	*Effect (low)*	*df*	χ^2^	*p*	*df*	χ^2^	*p*	*Effect (larger)*
*S. pulchra*	7	99.4	**<0.001**	1	1.44	0.23	Increase	7	7.30	0.40	1	36.9	**<0.001**	**Decrease**
*B. diandrus*	7	125.0	**<0.001**	1	0.40	0.53	Increase	7	6.87	0.44	1	13.2	**<0.001**	**Decrease**

Note

The watering treatment “effect” column indicates the effect of the low versus high treatment on emergence time. The seed mass “effect” column indicates the effect of increasing seed mass on emergence time. Significant effects (*p* < 0.05) are highlighted in bold.

**Table 2:** Results of GLMMs evaluating the effects of population, watering treatment, population × watering treatment interaction, and seed mass on individual seed outcomes (persistence, emergence, and mortality) in *Stipa pulchra* and *Bromus diandrus*

	Population			Watering treatment				Population × watering treatment			Seed mass			
	*df*	LRT	*p*	*df*	LRT	*p*	*Effect (low)*	*df*	LRT	*p*	*df*	LRT	*p*	*Effect (larger)*
*S. pulchra*														
Persistence	7	30.3	**<0.001**	1	3.81	0.051	Increase	7	3.59	0.83	1	6.38	**0.012**	**Decrease**
Emergence	7	54.1	**<0.001**	1	3.87	**0.049**	**Decrease**	7	8.34	0.30	1	119.2	**<0.001**	**Increase**
Mortality	7	58.9	**<0.001**	1	1.40	0.24	Increase	7	9.73	0.20	1	106.6	**<0.001**	**Decrease**
*B. diandrus*														
Persistence	7	64.6	**<0.001**	1	5.74	**0.017**	**Decrease**	7	5.73	0.57	1	4.06	**0.044**	**Decrease**
Emergence	7	73.0	**<0.001**	1	0.03	0.85	Increase	7	6.37	0.50	1	43.5	**<0.001**	**Increase**
Mortality	7	37.2	**<0.001**	1	8.09	**0.004**	**Increase**	7	4.99	0.66	1	41.9	**<0.001**	**Decrease**

Note

The watering treatment “effect” column indicates the effect of the low versus high treatment on the probability of each seed outcome. The seed mass “effect” column indicates the effect of increasing seed mass on each seed outcome. We evaluated the significance of model terms using likelihood ratio tests (LRT). Significant effects (*p* < 0.05) are highlighted in bold.

**Figure 3:**
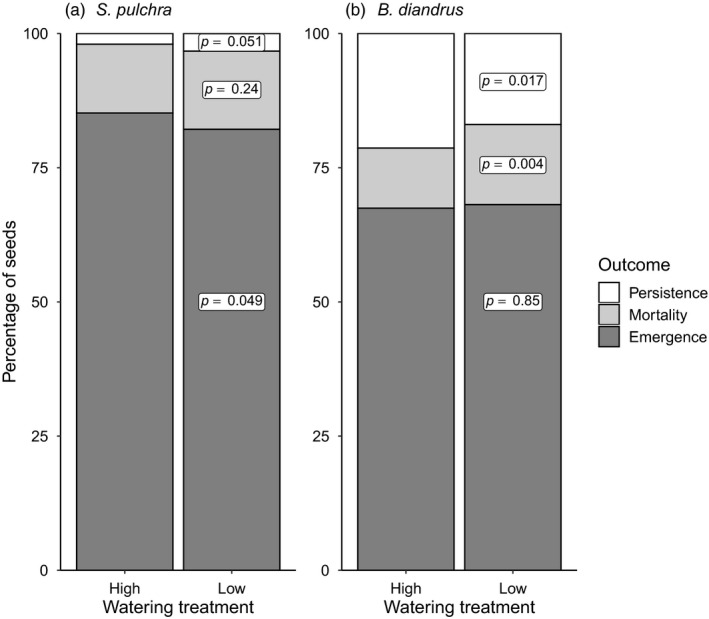
Influence of watering treatment on the outcomes of individual seeds in *Stipa pulchra* (a) and *Bromus diandrus* (b). Percentages are based on the raw values of all seeds pooled across source populations. *p* values are for watering treatment main effects in GLMMs (see Table 2)

Seed persistence, but not emergence time, responded plastically to watering in both focal species. Watering treatment did not significantly affect emergence time in either species (*S. pulchra*: *p* = 0.23; *B. diandrus*: *p* = 0.53; Table 1), and population × watering treatment interactions for emergence time were not significant in either species (*S. pulchra*: *p* = 0.40; *B. diandrus*: *p* = 0.44; Table 1). In *S. pulchra*, the probability of seed persistence was marginally significantly higher in the low watering treatment (*p* = 0.051; Table 2; Figure 3a), resulting from a lower probability of emergence (*p* = 0.049; Table 2; Figure 3a). In contrast, *B. diandrus* seeds were less likely to persist in the low watering treatment (*p* = 0.017; Table 2; Figure 3b), resulting from higher mortality (*p* = 0.004; Table 2; Figure 3b). In both species, population × watering treatment interactions were not significant for the probability of persistence (*S. pulchra*: *p* = 0.83; *B. diandrus*: *p* = 0.57; Table 2), emergence (*S. pulchra*: *p* = 0.30; *B. diandrus*: *p* = 0.50; Table 2), or mortality (*S. pulchra*: *p* = 0.20; *B. diandrus*: *p* = 0.66; Table 2).

### 3.2 Trade‐off between within‐year emergence speed and potential among‐year emergence spread

**Figure 4:**
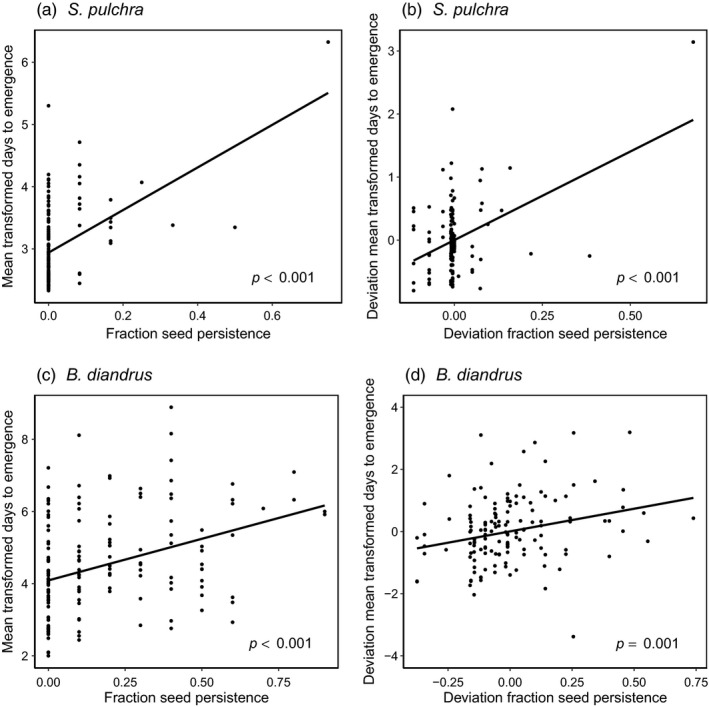
Scatter plots of seed persistence fraction in relation to emergence time in *Stipa pulchra* (a,b) and *Bromus diandrus* (c,d). Points in (a,c) represent values of mean transformed days to emergence and seed persistence fraction in maternal lines. Points in (b,d) represent the deviation of mean values in maternal lines from their respective source population means

As hypothesized, across all maternal lines larger fractions of persistent seeds were associated with later mean emergence in both species (*S. pulchra*: χ^2^(1) = 34.1, *p* < 0.001; *B. diandrus*: χ^2^(1) = 11.4, *p* < 0.001; Figure 4a,c), indicating a trade‐off between the speed of emergence within years and the potential for spreading emergence among years. In both species, there was a significant positive relationship between the deviations of mean maternal line emergence timing trait values from their respective population means (*S. pulchra*: χ^2^(1) = 31.9, *p* < 0.001; *B. diandrus*: χ^2^(1) = 10.5, *p* = 0.001; Figure 4b,d). Thus, the positive relationship between seed persistence fraction and mean emergence time occurred within populations and independently of covariation between population means. In *S. pulchra*, this positive association between seed persistence fraction and mean emergence time was strongly influenced by one maternal line from Stunt Ranch with very high seed persistence, which when excluded resulted in the relationship becoming no longer statistically significant (Cook's D > 7 for models of both original values and deviation values). Across all maternal lines, results were qualitatively the same after controlling for variation in emergence timing associated with seed mass (*S. pulchra*: χ^2^(1) = 33.4, *p* < 0.001; *B. diandrus*: χ^2^(1) = 10.4, *p* = 0.001), indicating that seed mass does not mediate the observed relationship between seed persistence fraction and emergence time.

### 3.3 Associations between emergence timing traits and site‐level aridity

**Figure 5:**
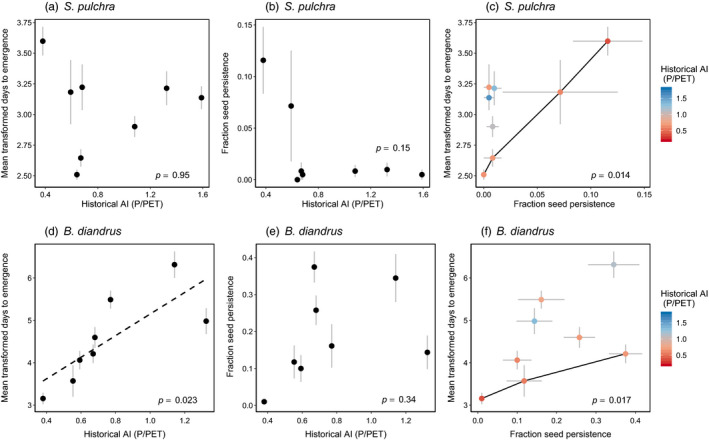
Associations between historical aridity of source populations and emergence timing traits in *Stipa pulchra* (a,b,c) and *Bromus diandrus* (d,e,f), for single traits (a,b,d,e) and trait combinations (c,f). Points represent values of mean transformed days to emergence and seed persistence fraction in source populations. Gray horizontal and vertical bars denote one standard error of the mean, where *n* is the number of maternal lines within each source population (see Table S2). In (c,f), black lines connect source populations that are on the Pareto front for earlier mean emergence and larger seed persistence fraction, and *p* values represent the results of permutation tests testing for differences in aridity between source populations on versus off the Pareto front. Lower values of historical AI indicate greater site aridity

In the native *S. pulchra*, the historical aridity of source populations did not significantly predict emergence time (*F*_(1, 6)_ = 0.004, *p* = 0.95; Figure 5a). The two most arid source populations exhibited the largest seed persistence fractions, but this trend was not statistically significant (*F*_(1, 6)_ = 2.76, *p* = 0.15; Figure 5b). The populations with trait combinations on the Pareto front for earlier emergence and larger seed persistence fraction experienced significantly more historically arid climates (*p* = 0.014; Figure 5c), consistent with constrained evolutionary responses to selection for earlier within‐year emergence and greater among‐year emergence spread. Relative aridity of collection year did not predict emergence time (*F*_(1, 6)_ = 0.35, *p* = 0.58) or seed persistence fraction (*F*_(1, 6)_ = 3.42, *p* = 0.11) and did not differ significantly between source populations that are on versus off the Pareto front (*p* = 0.20).

In the exotic *B. diandrus,* greater historical site aridity of source populations was associated with earlier emergence (*F*_(1, 6)_ = 9.19, *p* = 0.023; Figure 5d) but historical aridity had no effect on seed persistence fraction (*F*_(1, 6)_ = 1.09, *p* = 0.34; Figure 5e). This is consistent with the interpretation that, where the duration of favorable conditions for plant growth is relatively short, natural selection favors earlier emergence. Source populations on the Pareto front for earlier emergence and larger seed persistence fraction experienced significantly more arid climates than the remaining populations (*p* = 0.017; Figure 5f), again consistent with constrained evolutionary responses to selection for earlier within‐year emergence and greater among‐year emergence spread. Relative aridity of collection year did not significantly predict emergence time (*F*_(1, 6)_ = 1.31, *p* = 0.30) or seed persistence fraction (*F*_(1, 6)_ = 0.42, *p* = 0.54) and did not differ significantly between source populations that are on versus off the Pareto front (*p* = 0.22).

## 4 DISCUSSION

The timing of emergence within and among years are key traits influencing fitness in seasonal environments (Donohue et al., 2010). Our results provide evidence of a trade‐off between within‐year emergence speed and potential among‐year emergence spread that can constrain adaptive evolution in each trait. We demonstrate that this trade‐off can result in emergence timing trait values across an environmental gradient that appear suboptimal when traits are considered individually but are in fact on the Pareto front when considered in combination. We also found that plasticity in emergence timing traits has the potential to alter the fitness costs associated with the evolutionary constraint imposed by the trade‐off by causing phenotypic shifts either closer to or further away from the apparent local optimum. Our findings highlight the importance of considering emergence timing both within and among years when evaluating their adaptive significance.

### 4.1 Trade‐off between within‐year emergence speed and potential among‐year emergence spread

Maternal lines of *S. pulchra* and *B. diandrus* with larger fractions of persistent seeds (i.e., potential among‐year emergence spread) emerged later (Figure 4), indicating a trade‐off that can constrain adaptive evolution. The observed trade‐off could result from variation among maternal lines in the conditions that enforce dormancy or in those that cause emergence in non‐dormant seeds (or both); determining the underlying mechanisms that generate the trade‐off was beyond the scope of this study. In *S. pulchra,* the observed trade‐off was not robust, as it was strongly influenced by a single maternal line that produced highly persistent seeds, collected from the second most arid site, Stunt Ranch. The weak support for a trade‐off between emergence speed and among‐year emergence spread in this species is likely a consequence of its low overall seed persistence (85% of the maternal lines sampled had 0 persistent seeds), which is consistent with theory predicting lower seed dormancy in perennial than in annual species because adult survival can buffer perennials against poor environmental conditions (Rees, 1994).

Within species, there may be genetic variation in both dormancy and germination requirements (Fernández‐Pascual et al., 2013; Gremer et al., 2020), but in some species, these attributes are also strongly influenced by environmental factors such as the conditions during seed maturation and the degree of maternal provisioning (Fernández‐Pascual et al., 2013; Galloway, 2001; Halpern, 2005; Platenkamp & Shaw, 1993). Our experiment used field‐collected seeds, and therefore environmental variation within and among source population sites in the year of seed collection likely contributed to variation in emergence timing traits among maternal lines. However, we found that, among collection sites, aridity anomalies in the collection year (a measure of local conditions before and during seed maturation) did not predict emergence timing traits. We also found that the association between mean emergence time and seed persistence fraction occurred independently of mean seed mass in maternal lines, suggesting that the trade‐off between within‐year emergence speed and among‐year emergence spread was not mediated by variation in maternal provisioning; however, we were unable to test for effects of parental environments that are unrelated to provisioning, such as epigenetic inheritance (Henderson & Jacobsen, 2007). Fernández‐Pascual et al. (2013), compared dormancy in seeds of *Centaurium somedanum* collected from separate wild populations to seeds collected from a second generation grown in the greenhouse, and found that differences in seed maturation environment in source populations did not mask genetically based differences in dormancy. Thus, despite the potential for variation in seed maturation environment and maternal provisioning within and among source populations to influence values of emergence timing traits in our experiment, our results are consistent with a genetic basis for the trade‐off between within‐year emergence speed and potential among‐year emergence spread.

A limitation of our experiment is that persistent seeds could not be assessed for emergence time or continued soil persistence because the tetrazolium assay is lethal. Therefore, further work is needed to characterize the within‐year emergence time of seeds that persist for one or more years, as this has implications for the strength of the trade‐off. For example, relatively earlier emergence of persistent seeds in subsequent years could result in a weaker trade‐off when assessed across years compared to within a single year. However, while previous studies have investigated changes in the probability of seed persistence and/or germination over multiple years and the resulting impacts on population dynamics (Kalisz & McPeek, 1992, 1993; Philippi, 1993), we are aware of no studies that have investigated the within‐year emergence time of persistent seeds.

Seeds perform numerous critical life history functions among which there are trade‐offs due to biophysical or selective constraints (Venable & Brown, 1988); thus, the observed trade‐off between within‐year emergence speed and potential among‐year emergence spread likely represents one of several axes of variation that interact to influence fitness. In particular, seed size strongly influences performance in multiple life history functions and is likely to interact with emergence timing traits. For example, larger seed size enhances survival and reproduction in less favorable environments (Larios et al., 2014; Metz et al., 2010), and this might be particularly advantageous in environments that most strongly select for emergence before the onset of reliably tolerable conditions in the early growing season (cf. Skálová et al., 2011; Wainwright et al., 2012). On the other hand, smaller seeds can survive longer in the soil, partly because they are more easily incorporated to greater depths which reduces rates of postdispersal seed predation (Bekker et al., 1998; Hulme, 1998). Thus, smaller seed size is likely to be particularly favorable in environments in which selection strongly favors greater among‐year emergence spread. Consistent with this, in both of the focal species in the current study, larger seeds were more likely to emerge and emerged earlier (although the observed trade‐off between emergence speed and persistence occurred independently of seed size).

### 4.2 Associations between emergence timing traits and site‐level aridity

Increasing aridity might select for either earlier emergence within years (Dickman et al., 2019; Sexton et al., 2011), greater spread of emergence among years (Petrů & Tielbörger, 2008; Venable & Brown, 1988), or both, which, given a trade‐off between the two, might lead to constrained adaptive evolution. In the native perennial *S. pulchra*, the mean value of neither emergence timing trait was significantly associated with historical aridity of source populations when considered individually (Figure 5a,b). Overall seed persistence was low in this species, but the two source populations with the largest seed persistence fractions experienced the most arid climates, consistent with this species experiencing higher probabilities of unfavorable growing seasons in these sites. In *B. diandrus*, increasing aridity of source populations was associated with earlier emergence (Figure 5d), consistent with stronger selection for earlier emergence in the most arid environments. In both focal species, of the populations sampled, those that were on the Pareto front for earlier emergence and larger seed persistence fraction experienced more historically arid climates (Figure 5c,f), consistent with constrained evolutionary responses to selection for both earlier within‐year emergence and greater among‐year emergence spread in more arid climates.

Several factors may determine which combinations of emergence timing trait values evolve in response to increasing aridity. Firstly, favored trait value combinations may depend in part on selective constraints imposed by life history strategy (Rees et al., 2006). As an annual, *B. diandrus* may experience relatively strong selection to emerge early, facilitating the completion of its life cycle before favorable conditions deteriorate. Consistent with this, Dickman et al. (2019) found that a severe drought in California resulted in the evolution of earlier emergence in the annual *Mimulus laciniatus*. Among Sonoran Desert annuals, species that emerge earlier have higher water use efficiency (Kimball et al., 2011), and such adaptations in postemergence traits will likely mitigate the cost of lower among‐year emergence in *B. diandrus* populations occupying more arid sites. We expect that this trend toward earlier emergence would not persist beyond some threshold of aridity; annual plant communities in desert ecosystems characterized by exceptionally high interannual variability in precipitation, and thus variance in fitness among years, typically have both high among‐year emergence spread and diversified emergence within years (e.g., Gremer et al., 2016). In *S. pulchra*, a perennial species that does not typically flower in its first year, increasing aridity might not always result in stronger selection for earlier emergence, and thus, the relative strength of selection for earlier emergence versus greater among‐year emergence spread may differ among populations to a greater extent than in annual counterparts. In addition to patterns of selection, the geometry of the Pareto front will influence the trait values that evolve (Maharjan et al., 2013; Sheftel et al., 2013; Shoval et al., 2012), but determining this was beyond the scope of this study. With only two focal species, we have limited ability to test factors that influence the combinations of within‐ and among‐year emergence timing that evolve in response to increased aridity, but we highlight this as an important avenue for better understanding the process and multi‐trait outcome of adaptation to variable environments.

Besides evolutionary responses to aridity, several factors may have influenced which source populations were on the Pareto front for earlier emergence and larger seed persistence fraction. Firstly, optimum germination temperatures or soil moisture requirements can covary with local climatic conditions across a species range (Cavieres & Arroyo, 2000; Clauss & Venable, 2000; Meyer & Monsen, 1991). Greenhouse conditions may have resulted in earlier and more complete emergence of seeds collected from source populations that evolved in climatic conditions similar to conditions in the greenhouse (Bewley et al., 2013). Greenhouse temperatures were more similar to conditions in more arid sites and thus may have contributed to the pattern of earlier emergence with increasing aridity in *B. diandrus*. However, because more complete germination lowers the fraction of seeds that can persist, optimum germination conditions alone would not explain the association between increased site‐level aridity and Pareto optimality for earlier emergence and larger seed persistence fraction that we observed in both focal species. Secondly, populations experiencing more arid climates tend to be closer to each other (e.g., at lower latitudes) and thus might experience more gene flow, resulting in greater phenotypic similarity among them (Garant et al., 2007). However, in both focal species, the high‐aridity source populations on the Pareto front exhibit considerable diversity in trait value combinations (earlier emergence with lower seed persistence and later emergence with higher seed persistence), which does not support gene flow as the key factor driving Pareto optimality. Furthermore, in *B. diandrus*, the Pareto front is occupied by the first, third, and seventh most southerly source populations, which are unlikely to be the most interconnected. Thirdly, greater resource accumulation by parental plants could increase the quality of offspring seeds and lead to increased performance in multiple functions simultaneously (i.e., the Y‐model of trade‐offs, Roff & Fairbairn, 2007). However, our results do not support this as the mechanism driving Pareto optimality for earlier emergence and larger seed persistence fraction. In both species, higher seed mass, which reflects greater parental provisioning, was associated with earlier emergence but a lower probability of persistence (due to a higher probability of emergence). Additionally, in both species, aridity anomalies in the year of seed collection, a potential measure of the relative favorability of growing conditions compared to long‐term means, were not significantly associated with the position of populations with respect to the Pareto front.

Results from studies of emergence timing traits across putatively similar environmental gradients are notably inconsistent (reviewed in Cochrane et al., 2015). For example, across aridity gradients in Israel, populations of *Helianthemum* species in more arid sites have faster and more complete germination than those in more mesic sites (Gutterman & Edine, 1988), whereas populations of the grasses *Avena sterilis* and *Hordeum spontaneum* in more arid sites exhibit higher dormancy than those in more mesic sites (Volis, 2012). However, such studies typically test for associations between environmental variables and a single emergence timing trait and are therefore unlikely to characterize scenarios in which selection is acting on both within‐ and among‐year emergence timing. Our results for *S. pulchra* in particular illustrate how Pareto optimality of trait combinations can provide an adaptive explanation for individual traits that vary substantially across sites experiencing putatively similar climates. To our knowledge, this is the first study in which Pareto optimality has been applied to correlated traits across an environmental gradient to detect signatures of constrained evolution.

In this study, we evaluated two focal traits—within‐year emergence speed and potential among‐year emergence spread—that each determines performance in a separate function influencing plant fitness (i.e., early growth and bet‐hedging). Pareto optimality can also be evaluated for fitness‐related functions influenced by multiple traits (e.g., dispersal ability controlled by seed mass, seed shape, plant height etc.) (see Sheftel et al., 2013; Shoval et al., 2012). In any case, evaluating Pareto optimality requires knowledge of how trait values determine performance in given fitness‐related functions (Shoval et al., 2012). Previous studies have revealed evolutionary constraints by directly quantifying selection on correlated traits and showing that the vector of selection is orthogonal to the direction of the correlation (e.g., Etterson & Shaw, 2001). Evaluating Pareto optimality is not a substitute for such studies, but rather represents an extension to studying clinal variation in individual traits that is likely to be particularly useful when selection cannot be easily measured directly. For example, the adaptive value of certain traits—such as among‐year emergence spread—may be determined over multiple years or decades, while lifetime fitness is difficult to estimate for long‐lived, iteroparous species like *S. pulchra*.

### 4.3 Plastic responses of emergence timing traits to watering

We observed contrasting plastic responses of emergence timing traits to watering in the two focal species. Watering treatment had no effect on mean emergence time in either species, likely because many seeds emerged in response to initial pulses that were the same across treatments. In *S. pulchra*, seed persistence was marginally significantly higher in the low watering treatment due to a lower probability of emergence (Figure 3a). The direction of this plastic response is concordant with the apparent selective effect of increasing source population aridity (i.e., earlier emergence and larger seed persistence fraction) and is therefore consistent with predictive plasticity that could reduce the costs of the evolutionary constraint imposed by the trade‐off between within‐year emergence speed and among‐year emergence spread (cf. Gremer et al., 2016). In *B. diandrus*, seed persistence decreased in the low watering treatment due to higher seed mortality (Figure 3b), potentially caused by faster seed aging in warmer soils with lower latent heat loss (Long et al., 2015). The direction of this plastic response opposed the apparent selective effect of increasing source population aridity (i.e., earlier emergence and larger seed persistence fraction) and might therefore increase the costs of the evolutionary constraint imposed by the trade‐off between within‐year emergence speed and among‐year emergence spread. However, the significant decrease in mean emergence time with increasing historical aridity in *B. diandrus* suggests that increasing aridity most strongly selects for earlier within‐year emergence (cf. Dickman et al., 2019; Gutterman & Edine, 1988; Sexton et al., 2011); therefore, this plastic response of seed persistence to drier conditions might have a limited negative impact on fitness. Plasticity in emergence timing traits did not differ among source populations in either focal species; this could reflect either consistent selection on plasticity across populations or constraints on the evolution of plasticity in our focal emergence timing traits in response to spatially heterogeneous selection. Together, our results suggest that co‐occurring species may differ in the extent to which plasticity alters the fitness costs associated with the evolutionary constraint imposed by the trade‐off between within‐year emergence speed and among‐year emergence spread.

## 5 CONCLUSIONS

The timing of emergence within and among years are associated traits that must be considered together when investigating adaptation to current and future environmental conditions. Evaluating each emergence timing trait individually may lead researchers to incorrectly characterize patterns of historical selection acting on them across environmental gradients, which will result in less accurate predictions of adaptive responses to environmental change. Pareto optimality has only recently been applied to biological phenotypes (e.g., Sheftel et al., 2013; Shoval et al., 2012), but we suggest that this provides a promising tool for understanding patterns of trait variation across environmental gradients and thus predicting adaptation to future environmental change.

## 6 DATA ARCHIVING STATEMENT

Data, metadata, and R code are available on the Open Science Framework: https://doi.org/10.17605/OSF.IO/S7428.

## CONFLICT OF INTEREST

None declared.

## AUTHOR CONTRIBUTIONS

JW and EEC conceived the ideas and designed the methodology. JW collected and analyzed the data and led the writing of the manuscript. All authors contributed critically to the drafts and gave final approval for publication.

REFERENCES

Alpert, P.
, 
Krichak, S. O.
, 
Shafir, H.
, 
Haim, D.
, & 
Osetinsky, I.
 (2008). Climatic trends to extremes employing regional modeling and statistical interpretation over the E. Mediterranean. Global and Planetary Change, 63(2–3), 163–170. 10.1016/j.gloplacha.2008.03.003

AOSA/SCST
. (2010). Tetrazolium Testing Handbook (A. L. Miller 2010 ed.). Association of Official Seed Analysts and the Society of Commercial Seed Technologists.

Arroyo, M. T.
, 
Chacon, P.
, & 
Cavieres, L. A.
 (2006). Relationship between seed bank expression, adult longevity and aridity in species of *Chaetanthera* (Asteraceae) in central Chile. Annals of Botany, 98(3), 591–600. 10.1093/aob/mcl134
16820409PMC2803561

Aviad, Y.
, 
Kutiel, H.
, & 
Lavee, H.
 (2004). Analysis of beginning, end, and length of the rainy season along a Mediterranean–arid climate transect for geomorphic purposes. Journal of Arid Environments, 59(1), 189–204. 10.1016/j.jaridenv.2004.01.013


Baldwin, B. G.
, 
Goldman, D. H.
, 
Keil, D. J.
, 
Patterson, R.
, 
Rosatti, T. J.
, & 
Wilken, D. H.
 (Eds.) (2012). The Jepson manual: Vascular plants of California, 2nd ed. University of California Press.

Bartolome, J. W.
 (1979). Germination and seedling establishment in California annual grassland. Journal of Ecology, 67(1), 273–281. 10.2307/2259350


Bartolome, J. W.
, & 
Gemmill, B.
 (1981). The ecological status of *Stipa pulchra* (Poaceae) in California. Madroño, 28(3), 172–184.

Beguería, S.
, & 
Vicente‐Serrano, S. M.
 (2017). SPEI: Calculation of the standardised precipitation‐evapotranspiration index. R Package Version 1.7. Retrieved from https://cran.r‐project.org/package=SPEI


Bekker, R. M.
, 
Bakker, J. P.
, 
Grandin, U.
, 
Kalamees, R.
, 
Milberg, P.
, 
Poschlod, P.
, 
Thompson, K.
, & 
Willems, J. H.
 (1998). Seed size, shape and vertical distribution in the soil: Indicators of seed longevity. Functional Ecology, 12, 834–842. 10.1046/j.1365-2435.1998.00252.x


Berg, N.
, & 
Hall, A.
 (2015). Increased interannual precipitation extremes over California under climate change. Journal of Climate, 28, 6324–6334. 10.1175/JCLI-D-14-00624.1


Bewley, J. D.
, 
Bradford, K. J. M.
, 
Hilhorst, H.
, & 
Nonogaki, H.
 (2013). Seeds: Physiology of development, germination and dormancy, 3rd ed. Springer.

Bontrager, M.
, & 
Angert, A. L.
 (2016). Effects of range‐wide variation in climate and isolation on floral traits and reproductive output of *Clarkia pulchella*
. American Journal of Botany, 103(1), 10–21. 10.3732/ajb.1500091
26362193

Caruso, C. M.
 (2004). The quantitative genetics of floral trait variation in *Lobelia*: Potential constraints on adaptive evolution. Evolution, 58(4), 732–740. 10.1111/j.0014-3820.2004.tb00406.x
15154549

Cavieres, L. A.
, & 
Arroyo, M. T. K.
 (2000). Seed germination response to cold stratification period and thermal regime in *Phacelia secunda* (Hydrophyllaceae): Altitudinal variation in the mediterranean Andes of central Chile. Plant Ecology, 149, 1–8. 10.1023/A:1009802806674


Clauss, M. J.
, & 
Venable, D. L.
 (2000). Seed germination in desert annuals: An empirical test of adaptive bet hedging. The American Naturalist, 155, 168–186. 10.1086/303314
10686159

Cochrane, A.
, 
Yates, C. J.
, 
Hoyle, G. L.
, & 
Nicotra, A. B.
 (2015). Will among‐population variation in seed traits improve the chance of species persistence under climate change?
Global Ecology and Biogeography, 24(1), 12–24. 10.1111/geb.12234


Colautti, R. I.
, & 
Barrett, S. C. H.
 (2013). Rapid adaptation to climate facilitates range expansion of an invasive plant. Science, 342, 364–366. 10.1126/science.1242121
24136968

Davidowitz, G.
 (2002). Does precipitation variability increase from mesic to xeric biomes?
Global Ecology and Biogeography, 11(2), 143–154. 10.1046/j.1466-822x.2002.00271.x


Deering, R. H.
, & 
Young, T. P.
 (2006). Germination speeds of exotic annual and native perennial grasses in California and the potential benefits of seed priming for grassland restoration. Grasslands, 16, 14–16.

Dickman, E. E.
, 
Pennington, L. K.
, 
Franks, S. J.
, & 
Sexton, J. P.
 (2019). Evidence for adaptive responses to historic drought across a native plant species range. Evolutionary Applications, 12(8), 1569–1582. 10.1111/eva.12803
31462915PMC6708426

Donohue, K.
, 
Rubio de Casas, R.
, 
Burghardt, L.
, 
Kovach, K.
, & 
Willis, C. G.
 (2010). Germination, postgermination adaptation, and species ecological ranges. Annual Review of Ecology, Evolution, and Systematics, 41(1), 293–319. 10.1146/annurev-ecolsys-102209-144715


Dyer, A. R.
, 
Fenech, A.
, & 
Rice, K. J.
 (2000). Accelerated emergence in interspecific competitive neighbourhoods. Ecology Letters, 3, 523–529. 10.1111/j.1461-0248.2000.00187.x


Edwards, B.
, 
Burghardt, L. T.
, 
Kovach, K. E.
, & 
Donohue, K.
 (2017). Canalization of seasonal phenology in the presence of developmental variation: Seed dormancy cycling in an annual weed. Integrative and Comparative Biology, 57(5), 1021–1039. 10.1093/icb/icx065
28992196

Etterson, J. R.
, & 
Shaw, R. G.
 (2001). Constraint to adaptive evolution in response to global warming. Science, 294, 151–154. 10.1126/science.1063656
11588260

Exposito‐Alonso, M.
, 
Vasseur, F.
, 
Ding, W.
, 
Wang, G.
, 
Burbano, H. A.
, & 
Weigel, D.
 (2018). Genomic basis and evolutionary potential for extreme drought adaptation in *Arabidopsis thaliana*
. Nature Ecology & Evolution, 2(2), 352–358. 10.1038/s41559-017-0423-0
29255303PMC5777624

Fernández‐Pascual, E.
, 
Jiménez‐Alfaro, B.
, 
Caujapé‐Castells, J.
, 
Jaén‐Molina, R.
, & 
Díaz, T. E.
 (2013). A local dormancy cline is related to the seed maturation environment, population genetic composition and climate. Annals of Botany, 112, 937–945. 10.1093/aob/mct154
23864001PMC3747807

Fournier‐Level, A.
, 
Korte, A.
, 
Cooper, M. D.
, 
Nordborg, M.
, 
Schmitt, J.
, & 
Wilczek, A. M.
 (2011). A map of local adaptation in *Arabidopsis thaliana*
. Science, 334, 86–89. 10.1126/science.1209271
21980109

Fox, J.
, & 
Weisberg, S.
 (2011). An R companion to applied regression, 2nd ed. Sage.

Galloway, L. F.
 (2001). The effect of maternal and paternal environments on seed characters in the herbaceous plant *Campanula americana* (Campanulaceae). American Journal of Botany, 88(5), 832–840. 10.2307/2657035
11353708

Garant, D.
, 
Forde, S. E.
, & 
Hendry, A. P.
 (2007). The multifarious effects of dispersal and gene flow on contemporary adaptation. Functional Ecology, 21(3), 434–443. 10.1111/j.1365-2435.2006.01228.x


Gremer, J. R.
, 
Chiono, A.
, 
Suglia, E.
, 
Bontrager, M.
, 
Okafor, L.
, & 
Schmitt, J.
 (2020). Variation in the seasonal germination niche across an elevational gradient: The role of germination cueing in current and future climates. American Journal of Botany, 107(2), 350–363. 10.1002/ajb2.1425
32056208

Gremer, J. R.
, 
Kimball, S.
, & 
Venable, D. L.
 (2016). Within‐and among‐year germination in Sonoran Desert winter annuals: Bet hedging and predictive germination in a variable environment. Ecology Letters, 19(10), 1209–1218. 10.1111/ele.12655
27515951

Gutterman, Y.
, & 
Edine, L.
 (1988). Variations in seed germination of *Helianthemum vesicarium* and *H. ventosum* seeds from populations of two different altitudes in the Negev highlands, Israel. Journal of Arid Environments, 15(3), 261–267. 10.1016/s0140-1963(18)31063-2


Halpern, S. L.
 (2005). Sources and consequences of seed size variation in *Lupinus perennis* (Fabaceae): Adaptive and non‐adaptive hypotheses. American Journal of Botany, 92(2), 205–213. 10.3732/ajb.92.2.205
21652397

Hamilton, J. G.
, 
Griffin, J. R.
, & 
Stromberg, M. R.
 (2002). Long‐term population dynamics of native *Nassella* (Poaceae) bunchgrasses in central California. Madroño, 49(4), 274–284.

Harradine, A. R.
 (1986). Seed longevity and seedling establishment of *Bromus diandrus* Roth. Weed Research, 26, 173–180. 10.1111/j.1365-3180.1986.tb00693.x


Heady, H. F.
 (1977). Valley grassland. In 
M. G.
Barbour
, & 
J.
Major
 (Eds.), Terrestrial vegetation in California (pp. 491–514). Wiley.

Henderson, I. R.
, & 
Jacobsen, S. E.
 (2007). Epigenetic inheritance in plants. Nature, 447, 418–424. 10.1038/nature05917
17522675

Hoffmann, A. A.
, & 
Sgro, C. M.
 (2011). Climate change and evolutionary adaptation. Nature, 470, 479–485. 10.1038/nature09670
21350480

Huang, X.
, 
Schmitt, J.
, 
Dorn, L.
, 
Griffith, C.
, 
Effgen, S.
, 
Takao, S.
, 
Koornneef, M.
, & 
Donohue, K.
 (2010). The earliest stages of adaptation in an experimental plant population: Strong selection on QTLS for seed dormancy. Molecular Ecology, 19, 1335–1351. 10.1111/j.1365-294X.2010.04557.x
20149097

Hulme, P. E.
 (1998). Post‐dispersal seed predation and seed bank persistence. Seed Science Research, 8(4), 513–519. 10.1017/s0960258500004487

IPCC
. (2013). Annex I: Atlas of global and regional climate projections. In 
G. J. van Oldenborgh
, 
M.
Collins
, 
J.
Arblaster
, 
J. H.
Christensen
, 
J.
Marotzke
, 
S. B.
Power
, 
M.
Rummukainen
, & 
T.
Zhou
 (Eds.). T. F. Stocker, D. Qin, G.‐K. Plattner, M. Tignor, S. K. Allen, J. Boschung, A. Nauels, Y. Xia, V. Bex & P. M. Midgley. (Series Eds.), Climate Change 2013: The Physical Science Basis. Contribution of Working Group I to the Fifth Assessment Report of the Intergovernmental Panel on Climate Change. Cambridge University Press.

Jump, A. S.
, & 
Peñuelas, J.
 (2005). Running to stand still: Adaptation and the response of plants to rapid climate change. Ecology Letters, 8(9), 1010–1020. 10.1111/j.1461-0248.2005.00796.x
34517682

Kalisz, S.
 (1986). Variable selection on the timing of germination in *Collinsia verna* (Scrophulariaceae). Evolution, 40(3), 479–491. 10.2307/2408571
28556331

Kalisz, S.
, & 
McPeek, M. A.
 (1992). Demography of an age‐structured annual: Resampled projection matrices, elasticity analyses, and seed bank effects. Ecology, 73(3), 1082–1093. 10.2307/1940182


Kalisz, S.
, & 
McPeek, M. A.
 (1993). Extinction dynamics, population growth and seed banks. Oecologia, 95, 314–320. 10.1007/BF00320982
28314004

Kimball, S.
, 
Angert, A. L.
, 
Huxman, T. E.
, & 
Venable, D. L.
 (2011). Differences in the timing of germination and reproduction relate to growth physiology and population dynamics of Sonoran Desert winter annuals. American Journal of Botany, 98(11), 1773–1781. 10.3732/ajb.1100034
22003177

Kleemann, S. G. L.
, & 
Gill, G. S.
 (2013). Seed dormancy and seedling emergence in ripgut brome (*Bromus diandrus*) populations in Southern Australia. Weed Science, 61(2), 222–229. 10.1614/ws-d-12-00083.1


Knapp, E. E.
, & 
Rice, K. J.
 (1998). Comparison of isozymes and quantitative traits for evaluating patterns of genetic variation in purple needlegrass (*Nassella pulchra*). Conservation Biology, 12(5), 1031–1041. 10.1046/j.1523-1739.1998.97123.x


Kon, K. F.
, & 
Blacklow, W. M.
 (1990). Polymorphism, outcrossing and polyploidy in *Bromus diandrus* and *B. rigidus*
. Australian Journal of Botany, 38(6), 609–618. 10.1071/BT9900609


Larios, L.
, 
Búrquez, A.
, 
Becerra, J. X.
, & 
Venable, D. L.
 (2014). Natural selection on seed size through the life cycle of a desert annual plant. Ecology, 95(11), 3213–3220. 10.1890/13-1965.1


Larson, S. R.
, 
Cartier, E.
, 
McCracken, C. L.
, & 
Dyer, D.
 (2001). Mode of reproduction and amplified fragment length polymorphism variation in purple needlegrass (*Nassella pulchra*): Utilization of natural germplasm sources. Molecular Ecology, 10, 1165–1177. 10.1046/j.1365-294X.2001.01267.x
11380875

Long, R. L.
, 
Gorecki, M. J.
, 
Renton, M.
, 
Scott, J. K.
, 
Colville, L.
, 
Goggin, D. E.
, 
Commander, L. E.
, 
Westcott, D. A.
, 
Cherry, H.
, & 
Finch‐Savage, W. E.
 (2015). The ecophysiology of seed persistence: A mechanistic view of the journey to germination or demise. Biological Reviews, 90, 31–59. 10.1111/brv.12095
24618017

Maharjan, R.
, 
Nilsson, S.
, 
Sung, J.
, 
Haynes, K.
, 
Beardmore, R. E.
, 
Hurst, L. D.
, 
Ferenci, T.
, & 
Gudelj, I.
 (2013). The form of a trade‐off determines the response to competition. Ecology Letters, 16(10), 1267–1276. 10.1111/ele.12159
23902419

Malmström, V. H.
 (1969). A new approach to the classification of climate. Journal of Geography, 68(6), 351–357. 10.1080/00221346908981131


Marañón, T.
, & 
Bartolome, J. W.
 (1989). Seed and seedling populations in two contrasted communities: Open grassland and oak (*Quercus agrifolia*) understory in California. Acta Oecologica, Oecologica Plantarum, 10(2), 147–158.

Mazer, S. J.
, 
Park, I. M.
, 
Kimura, M.
, 
Maul, E. M.
, 
Yim, A. M.
, 
Peach, K.
, & 
Bonser, S.
 (2020). Mating system and historical climate conditions affect population mean seed mass: Evidence for adaptation and a new component of the selfing syndrome in *Clarkia*
. Journal of Ecology, 108(4), 1523–1539. 10.1111/1365-2745.13338


Metz, J.
, 
Lampei, C.
, 
Bäumler, L.
, 
Bocherens, H.
, 
Dittberner, H.
, 
Henneberg, L.
, 
Meaux, J.
, & 
Tielbörger, K.
 (2020). Rapid adaptive evolution to drought in a subset of plant traits in a large‐scale climate change experiment. Ecology Letters, 23(11), 1643–1653. 10.1111/ele.13596
32851791

Metz, J.
, 
Liancourt, P.
, 
Kigel, J.
, 
Harel, D.
, 
Sternberg, M.
, & 
Tielbörger, K.
 (2010). Plant survival in relation to seed size along environmental gradients: A long‐term study from semi‐arid and Mediterranean annual plant communities. Journal of Ecology, 98(3), 697–704. 10.1111/j.1365-2745.2010.01652.x


Meyer, S. E.
, & 
Monsen, S. B.
 (1991). Habitat‐correlated variation in mountain big sagebrush (*Artemisia tridentata* ssp. *vaseyana*) seed germination patterns. Ecology, 72(2), 739–742. 10.2307/2937214


Mordecai, E. A.
 (2012). Soil moisture and fungi affect seed survival in California grassland annual plants. PLoS One, 7(6), e39083. 10.1371/journal.pone.0039083
22720037PMC3373626

Munson, S. M.
, & 
Sher, A. A.
 (2015). Long‐term shifts in the phenology of rare and endemic Rocky Mountain plants. American Journal of Botany, 102(8), 1268–1276. 10.3732/ajb.1500156
26290550

Petrů, M.
, & 
Tielbörger, K.
 (2008). Germination behaviour of annual plants under changing climatic conditions: Separating local and regional environmental effects. Oecologia, 155(4), 717–728. 10.1007/s00442-007-0955-0
18204993

Philippi, T.
 (1993). Bet‐hedging germination of desert annuals: Beyond the first year. The American Naturalist, 142(3), 474–487. 10.1086/285550
19425987

Pinheiro, J.
, 
Bates, D.
, 
DebRoy, S.
, 
Sarkar, D.
, & R Core Team
. (2019). nlme: Linear and nonlinear mixed effects models. R package version 3.1‐140. Retrieved from https://cran.r‐project.org/package=nlme


Platenkamp, G. A.
, & 
Shaw, R. G.
 (1993). Environmental and genetic maternal effects on seed characters in *Nemophila menziesii*
. Evolution, 47(2), 540–555. 10.1111/j.1558-5646.1993.tb02112.x
28568726

Pratt, J. D.
, & 
Mooney, K. A.
 (2013). Clinal adaptation and adaptive plasticity in *Artemisia californica*: Implications for the response of a foundation species to predicted climate change. Global Change Biology, 19(8), 2454–2466. 10.1111/gcb.12199
23505064
R Core Team
. (2019). R: A language and environment for statistical computing. Vienna, Austria: R Foundation for Statistical Computing. Retrieved from https://www.r‐project.org/


Rees, M.
 (1994). Delayed germination of seeds: A look at the effects of adult longevity, the timing of reproduction, and population age/stage structure. The American Naturalist, 144(1), 43–64. 10.1086/285660


Rees, M.
, 
Childs, D. Z.
, 
Metcalf, C. J.
, 
Rose, K. E.
, 
Sheppard, A. W.
, & 
Grubb, P. J.
 (2006). Seed dormancy and delayed flowering in monocarpic plants: Selective interactions in a stochastic environment. The American Naturalist, 168(2), E53–E71. 10.1086/505762
16874623

Rizopoulos, D.
 (2019). GLMMadaptive: Generalized linear mixed models using adaptive gaussian quadrature. R package version 0.6‐0. Retrieved from https://cran.r‐project.org/package=GLMMadaptive


Roff, D. A.
, & 
Fairbairn, D. J.
 (2007). The evolution of trade‐offs: Where are we?
Journal of Evolutionary Biology, 20(2), 433–447. 10.1111/j.1420-9101.2006.01255.x
17305809

Roocks, P.
 (2016). Computing Pareto frontiers and database preferences with the rPref package. The R Journal, 8(2), 393–404. 10.32614/RJ-2016-054


Saatkamp, A.
, 
Affre, L.
, 
Dutoit, T.
, & 
Poschlod, P.
 (2011). Germination traits explain soil seed persistence across species: The case of Mediterranean annual plants in cereal fields. Annals of Botany, 107(3), 415–426. 10.1093/aob/mcq255
21224268PMC3043931

Seager, R.
, 
Ting, M.
, 
Held, I.
, 
Kushnir, Y.
, 
Lu, J.
, 
Vecchi, G.
, 
Huang, H.‐P.
, 
Harnik, N.
, 
Leetmaa, A.
, 
Lau, N.‐C.
, 
Li, C.
, 
Velez, J.
, & 
Naik, N.
 (2007). Model projections of an imminent transition to a more arid climate in southwestern North America. Science, 316, 1181–1184. 10.1126/science.1139601
17412920

Sexton, J. P.
, 
Strauss, S. Y.
, & 
Rice, K. J.
 (2011). Gene flow increases fitness at the warm edge of a species’ range. Proceedings of the National Academy of Sciences USA, 108(28), 11704–11709. 10.1073/pnas.1100404108
PMC313625221709253

Sheftel, H.
, 
Shoval, O.
, 
Mayo, A.
, & 
Alon, U.
 (2013). The geometry of the Pareto front in biological phenotype space. Ecology and Evolution, 3(6), 1471–1483. 10.1002/ece3.528
23789060PMC3686184

Shoval, O.
, 
Sheftel, H.
, 
Shinar, G.
, 
Hart, Y.
, 
Ramote, O.
, 
Mayo, A.
, 
Dekel, E.
, 
Kavanagh, K.
, & 
Alon, U.
 (2012). Evolutionary trade‐offs, Pareto optimality, and the geometry of phenotype space. Science, 336, 1157–1160. 10.1126/science.1217405
22539553

Simons, A. M.
, & 
Johnston, M. O.
 (2006). Environmental and genetic sources of diversification in the timing of seed germination: Implications for the evolution of bet hedging. Evolution, 60(11), 2280–2292. 10.1111/j.0014-3820.2006.tb01865.x
17236421

Skálová, H.
, 
Moravcová, L.
, & 
Pyšek, P.
 (2011). Germination dynamics and seedling frost resistance of invasive and native *Impatiens* species reflect local climatic conditions. Perspectives in Plant Ecology, Evolution and Systematics, 13(3), 173–180. 10.1016/j.ppees.2011.03.005


Stearns, S.
, 
de Jong, G.
, & 
Newman, B.
 (1991). The effects of phenotypic plasticity on genetic correlations. Trends in Ecology & Evolution, 6(4), 122–126. 10.1016/0169-5347(91)90090-K
21232440

Stromberg, M. R.
, & 
Griffin, J. R.
 (1996). Long‐term patterns in coastal California grasslands in relation to cultivation, gophers, and grazing. Ecological Applications, 6(4), 1189–1211. 10.2307/2269601


Thornthwaite, C. W.
 (1948). An approach toward a rational classification of climate. Geographical Review, 38(1), 55–94. 10.2307/210739


Tielbörger, K.
, 
Petrů, M.
, & 
Lampei, C.
 (2012). Bet‐hedging germination in annual plants: A sound empirical test of the theoretical foundations. Oikos, 121, 1860–1868. 10.1111/j.1600-0706.2011.20236.x


Tilley, D.
, 
Dyer, D.
, & 
Anderson, J.
 (2009). Plant guide: Purple needlegrass, Nassella pulchra (Hitchc.) Barkworth. USDA, Natural Resource Conservation Service (NRCS).

Torres‐Martínez, L.
, 
Weldy, P.
, 
Levy, M.
, & 
Emery, N. C.
 (2017). Spatiotemporal heterogeneity in precipitation patterns explain population‐level germination strategies in an edaphic specialist. Annals of Botany, 119(2), 253–265. 10.1093/aob/mcw161
27551027PMC5321057

Venable, D. L.
, & 
Brown, J. S.
 (1988). The selective interactions of dispersal, dormancy and seed size as adaptations for reducing risk in variable environments. The American Naturalist, 131(3), 360–384. 10.1086/284795


Verdú, M.
, & 
Traveset, A.
 (2005). Early emergence enhances plant fitness: A phylogenetically controlled meta‐analysis. Ecology, 86(6), 1385–1394. 10.1890/04-1647


Volis, S.
 (2012). Demographic consequences of delayed germination in two annual grasses from two locations of contrasting aridity. Perspectives in Plant Ecology, Evolution and Systematics, 14(5), 335–340. 10.1016/j.ppees.2012.07.002


Wadgymar, S. M.
, 
Daws, S. C.
, & 
Anderson, J. T.
 (2017). Integrating viability and fecundity selection to illuminate the adaptive nature of genetic clines. Evolution Letters, 1(1), 26–39. 10.1002/evl3.3
30283636PMC6121800

Wainwright, C. E.
, & 
Cleland, E. E.
 (2013). Exotic species display greater germination plasticity and higher germination rates than native species across multiple cues. Biological Invasions, 15(10), 2253–2264. 10.1007/s10530-013-0449-4


Wainwright, C. E.
, 
Wolkovich, E. M.
, & 
Cleland, E. E.
 (2012). Seasonal priority effects: Implications for invasion and restoration in a semi‐arid system. Journal of Applied Ecology, 49, 234–241. 10.1111/j.1365-2664.2011.02088.x


Walck, J. L.
, 
Hidayati, S. N.
, 
Dixon, K. W.
, 
Thompson, K.
, & 
Poschlod, P.
 (2011). Climate change and plant regeneration from seed. Global Change Biology, 17, 2145–2161. 10.1111/j.1365-2486.2010.02368.x


Waterton, J.
, & 
Cleland, E. E.
 (2016). Trade‐off between early emergence and herbivore susceptibility mediates exotic success in an experimental California plant community. Ecology and Evolution, 6(24), 8942–8953. 10.1002/ece3.2610
28035282PMC5192797

Yoon, J. H.
, 
Wang, S. Y.
, 
Gillies, R. R.
, 
Kravitz, B.
, 
Hipps, L.
, & 
Rasch, P. J.
 (2015). Increasing water cycle extremes in California and in relation to ENSO cycle under global warming. Nature Communications, 6, 8657. 10.1038/ncomms9657
PMC463989826487088

Young, J. A.
, 
Evans, R. A.
, 
Raguse, C. A.
, & 
Larson, J. R.
 (1981). Germinable seeds and periodicity of germination in annual grasslands. Hilgardia, 49(2), 1–37. 10.3733/hilg.v49n02p037


## Supporting information

Table S1‐S2
