# Supplementary material for: Trade‐off drives Pareto optimality of within‐ and among‐year emergence timing in response to increasing aridity
Source: Evol Appl. 2020 Nov 6;14(3):658–73. doi: 10.1111/eva.13145 (PMC7980269; doi:10.1111/eva.13145)
Supplement: Supplementary file 1 — Table S1‐S2 [file EVA-14-658-s001.docx]

**Table S1.** Source population climate summaries. Historical climate variables are for the years 1985 – 2014 and collection year variables are for May 2014 – April 2015.

| Site | Latitude | Longitude | Historical mean annual precipitation (mm) | Historical mean annual temperature (°C) | Historical annual PET (mm) | Historical aridity index | Collection year precipitation (mm) | Collection year mean temperature (°C) | Collection year PET (mm) | Collection year aridity index | Collection year deviation aridity index |
| --- | --- | --- | --- | --- | --- | --- | --- | --- | --- | --- | --- |
| A) Hopland | 39.02 | -123.09 | 997 | 14.79 | 758 | 1.315 | 835 | 16.58 | 873 | 0.957 | -0.357 |
| B) Bodega | 38.42 | -123.10 | 1087 | 13.35 | 683 | 1.590 | 797 | 14.64 | 759 | 1.050 | -0.540 |
| C) Quail Ridge | 38.48 | -122.14 | 806 | 14.40 | 746 | 1.081 | 578 | 15.85 | 834 | 0.694 | -0.387 |
| D) Jepson Prairie | 38.27 | -121.83 | 520 | 15.91 | 800 | 0.650 | 342 | 17.33 | 892 | 0.384 | -0.267 |
| E) Younger Lagoon | 36.95 | -122.07 | 764 | 13.08 | 669 | 1.141 | 657 | 15.21 | 808 | 0.812 | -0.329 |
| F) Fort Ord | 36.69 | -121.77 | 379 | 13.76 | 684 | 0.554 | 316 | 15.23 | 777 | 0.406 | -0.147 |
| G) Hastings | 36.38 | -121.57 | 559 | 14.43 | 725 | 0.771 | 395 | 16.43 | 853 | 0.464 | -0.307 |
| H) Landels-Hill | 36.07 | -121.59 | 944 | 14.53 | 713 | 1.324 | 573 | 16.33 | 829 | 0.691 | -0.633 |
| I) Kenneth S. Norris | 35.53 | -121.08 | 459 | 13.67 | 675 | 0.680 | 318 | 15.77 | 813 | 0.391 | -0.290 |
| J) Sedgwick | 34.69 | -120.04 | 541 | 16.62 | 807 | 0.670 | 245 | 17.93 | 900 | 0.272 | -0.398 |
| K) Coal Oil Point | 34.42 | -119.88 | 469 | 15.25 | 733 | 0.640 | 220 | 16.74 | 834 | 0.263 | -0.377 |
| L) Stunt Ranch | 34.09 | -118.66 | 497 | 17.68 | 839 | 0.593 | 221 | 19.57 | 979 | 0.225 | -0.367 |
| M) Elliott Chaparral | 32.89 | -117.09 | 316 | 17.63 | 832 | 0.380 | 200 | 19.48 | 979 | 0.205 | -0.176 |

**Table S2.** Number of maternal lines in each source population in which, across both watering treatments, more than 50% of seeds were scored as “viable” (i.e., either emerged or remained persistent). We excluded from statistical analyses all maternal lines not meeting this threshold value and excluded source populations with fewer than 10 maternal lines meeting this viability threshold. Source populations included in statistical analyses are shown in bold. We did not collect *B. diandrus* seeds from Hopland, Bodega, Quail Ridge, Jepson Prairie and Coal Oil Point.

| Site | Maternal lines meeting 50% viable seeds threshold (out of 20) | |
| --- | --- | --- |
|  | *S. pulchra* | *B. diandrus* |
| Hopland | 6 | -- |
| Bodega | **17** | -- |
| Quail Ridge | **20** | -- |
| Jepson Prairie | 3 | -- |
| Younger Lagoon | 4 | **20** |
| Fort Ord | 8 | **17** |
| Hastings | 5 | **18** |
| Landels-Hill | **17** | **16** |
| Kenneth S. Norris | **17** | **19** |
| Sedgwick | **20** | **20** |
| Coal Oil Point | **20** | -- |
| Stunt Ranch | **14** | **20** |
| Elliott Chaparral | **18** | **20** |
